# Functional Variants Surrounding Endothelin 2 Are Associated With *Mycobacterium avium* Subspecies *paratuberculosis* Infection

**DOI:** 10.3389/fvets.2021.625323

**Published:** 2021-05-05

**Authors:** Jennifer N. Kiser, Zeping Wang, Ricardo Zanella, Erik Scraggs, Mahesh Neupane, Bonnie Cantrell, Curtis P. Van Tassell, Stephen N. White, Jeremy F. Taylor, Holly L. Neibergs

**Affiliations:** ^1^Department of Animal Sciences, Washington State University, Pullman, WA, United States; ^2^Animal Genomics and Improvement Laboratory, United States Department of Agriculture, Agricultural Research Service, Beltsville, MD, United States; ^3^Animal Disease Research, United States Department of Agriculture, Agricultural Research Service, Pullman, WA, United States; ^4^Department of Veterinary Microbiology and Pathology, Washington State University, Pullman, WA, United States; ^5^Center for Reproductive Biology, Washington State University, Pullman, WA, United States; ^6^Division of Animal Sciences, University of Missouri, Columbia, MO, United States

**Keywords:** paratubercolosis, dairy cattle, EDN2, functional variant, functional assays

## Abstract

Bovine paratuberculosis, caused by *Mycobacterium avium* subspecies *paratuberculosis* (*MAP*), continues to impact the dairy industry through increased morbidity, mortality, and lost production. Although genome-wide association analyses (GWAAs) have identified loci associated with susceptibility to *MAP*, limited progress has been made in identifying mutations that cause disease susceptibility. A 235-kb region on *Bos taurus* chromosome 3 (BTA3), containing a 70-kb haplotype block surrounding endothelin 2 (*EDN2*), has previously been associated with the risk of *MAP* infection. *EDN2* is highly expressed in the gut and is involved in intracellular calcium signaling and a wide array of biological processes. The objective of this study was to identify putative causal mutations for disease susceptibility in the region surrounding *EDN2* in Holstein and Jersey cattle. Using sequence data from 10 Holstein and 10 Jersey cattle, common variants within the 70-kb region containing *EDN2* were identified. A custom SNP genotyping array fine-mapped the region using 221 Holstein and 51 Jersey cattle and identified 17 putative causal variants (*P* < 0.01) located in the 5′ region of *EDN2* and a SNP in the 3′ UTR (*P* = 0.00009) associated with *MAP* infection. MicroRNA interference assays, mRNA stability assays, and electrophoretic mobility shift assays were performed to determine if allelic changes at each SNP resulted in differences in *EDN2* stability or expression. Two SNPs [*rs109651404* (G/A) and *rs110287192* (G/T)] located within the promoter region of *EDN2* displayed differential binding affinity for transcription factors in binding sequences harboring the alternate SNP alleles. The luciferase reporter assay revealed that the transcriptional activity of the *EDN2* promoter was increased (*P* < 0.05) with the A allele for *rs109651404* and the G allele for *rs110287192*. These results suggest that the variants *rs109651404* and *rs110287192* are mutations that alter transcription and thus may alter susceptibility to *MAP* infection in Holstein and Jersey cattle.

## Introduction

Paratuberculosis, also referred to as Johne's disease, is an infectious and incurable disease that afflicts many wild and domestic ruminant species, including cattle, sheep, goats, and deer. Caused by *Mycobacterium avium* ssp. *paratuberculosis* (*MAP*), most cattle with bovine paratuberculosis initially present with decreased production and progress to severe diarrhea, emaciation, and eventually death. With no effective vaccines available for the disease and limited treatment options for livestock, many countries rely on voluntary or mandatory management and prevention programs to help producers limit the spread of infection both within and between herds (1). The financial burden incurred by producers due to bovine paratuberculosis is substantial. In the U.S. dairy industry alone, *MAP* infections result in an estimated $1.5 billion annual loss stemming from decreases in production (2).

While the economic impact of bovine paratuberculosis is substantial, the situation is made worse by a lack of sensitive diagnostic tests and limited treatment options. There are multiple diagnostic tests available to test for *MAP* infection including polymerase chain reaction (PCR) analyses of tissues or feces; enzyme-linked immunosorbent assays (ELISAs) for serum, milk, or fecal samples; and culturing of tissue or feces, all of which have similar specificity for *MAP* (3, 4). However, the sensitivity of these diagnostic methods varies. For example, McKenna et al. (5) reported that when 160 cattle identified as *MAP* positive by tissue culture were tested for *MAP* infection by fecal ELISAs, the ELISAs only identified 6.9% (*n* = 11) to 16.9% (*n* = 27) of the cattle as being *MAP* positive. Among the various diagnostic methods available for MAP testing, tissue culturing is considered the gold standard (6, 7). However, tissue culturing is a time-consuming method and studies have shown that quantitative PCR of tissue samples is equally effective in identifying MAP-positive animals and considerably faster (8), making the use of the two diagnostic methods comparable. The differences among some testing methods have likely contributed to the lack of success in curbing the spread of the disease.

The identification of genetic markers from studies with high-quality phenotypes can be implemented to the population at large to help to reduce the prevalence of the disease. Previous studies have identified loci associated with disease susceptibility that could be used to help producers select less susceptible cattle. These include candidate gene studies (9–14) and genome-wide association studies (11, 15–24). To date, there have been few loci that have been associated with *MAP* in more than one study, which may be due to differences in *MAP* diagnostic testing sensitivity, genotyping densities, and disease phenotype definition (22). However, some concordance among associations has been found on *Bos taurus* chromosome 1 (BTA1) (15, 19), BTA12 (16, 19), and BTA3 (15, 25). The overlapping regions between studies on BTA1 and BTA12 do not harbor any positional candidate genes within 50 kb of the associated loci, but the region on BTA3 harbored a 70 kb haplotype block that is located near endothelin 2 (*EDN2*) and HIVEP zinc finger 3 (*HIVEP3*).

In a study by Zanella et al. (18), the original 235-kb candidate region on BTA3 was further refined to 10.6 kb (104,738,280 to 104,748,953 bp on ARS-UCD 1.2) near *EDN2*. The objective of the current study was to validate and refine the 10.6-kb region and to further identify putative causal mutations within this 10.6-kb region associated with susceptibility to *MAP* tissue infection in both Holstein and Jersey cattle. To determine if loci located in the 10.6-kb region were associated with differences between *MAP*-positive and *MAP*-negative cattle, microRNA interference assays, mRNA stability assays, electrophoretic mobility shift assays (EMSAs), and an association analysis were utilized.

## Materials and Methods

### Study Populations

Sample collection and *MAP* diagnostic methods for the Holstein population used in this study have been described in detail in previous *MAP* studies (15, 18, 22, 25, 26). Briefly, 245 Holstein cows from four herds in the Eastern United States had tissue and fecal samples collected at slaughter. To determine if the animals were *MAP* tissue infected, *MAP* was cultured from tissues taken from the ileum, ileo-cecal valve, and two adjacent ileo-cecal lymph nodes using the method described by Whitlock et al. (3). Samples with colony-forming units (cfus) of *MAP* > 0 were classified as *MAP* infected. After testing, 94 cattle were classified as cases, and 138 cattle were classified as controls. An additional 13 cattle were not tissue cultured and were removed from further analyses. The mean ages of cases (60.7 months) and controls (58.5 months) did not differ (*P* = 0.44).

As with the Holstein population, sample collection and diagnostic testing methods of the Jersey population have also been previously described (22). The Jersey population consisted of 48 cows from an Oregon dairy and 9 Jersey steers from a dairy in Pennsylvania. Briefly, fecal and tissue samples from ileum and ileo-cecal lymph nodes were harvested at slaughter, and determination of the infection status of the animals was conducted using the AgPath-ID^tm^ real-time qPCR assay using 300 mg of tissue sample (ileo-cecal lymph node and ileum) for the Northern Oregon animals. Quantitative PCR results were also confirmed in a subset of samples with qPCR results from the Washington State University Veterinary Diagnostic Laboratory. All qPCR diagnostic testing was performed in triplicate. Animals with *MAP* DNA copies/μl >1 in at least one of the tissues were considered *MAP* infected. After testing, 16 cattle were classified as cases and 41 were classified as controls. The mean age of cases (46.2 months) did not differ from the mean age of controls (52.6 months) (*P* = 0.48).

### Genotyping and Quality Assurance

To develop a custom fine-mapping array, single-nucleotide polymorphisms (SNPs) within the previously defined region on BTA3 (104,677,793 to 104,748,725 bp on ARS-UCD 1.2) were identified using the whole genome sequences of 10 Holstein and 10 Jersey cattle previously sequenced for a different study (*MAP* status unknown) (Animal Genomics and Improvement Laboratory, United States Department of Agriculture). The non-coding region between *HIVEP3* and *EDN2* was observed to be highly conserved when compared to six species (human, macaque, horse, elephant, dog, and dolphin) with blocks of conservation that exceeded the conservation of the coding regions of *HIVEP3* and *EDN2*. Non-coding regions commonly contain regulatory elements such as enhancers and promoters that may be evolutionarily conserved across species (27). Conserved sequences across species commonly indicate regions constrained against mutation due to the functional consequences of such a variant. Given the lack of annotation of the bovine genome assembly, sequence comparison across species allows for the identification of DNA sequences previously identified as regulatory elements in other, better annotated species like humans and identify conserved sequences that typically have functional roles. This region between *EDN2* and *HIVEP3* contains a multitude of transcription factor binding sites and over 100 putative functional modifications that modify the binding of transcription factors based on *in silico* analysis of the observed DNA variants. From the whole genome sequence data, 528 SNPs with a median spacing of 132 bp were identified in the candidate region. From these 528 SNPs, 96 were incorporated in a custom 96-SNP assay (Illumina, San Diego, CA). These 96 SNPs were selected based on a lack of repetitive regions surrounding the SNP, their nucleotide location based on the UMD 3.0 assembly, high minor allele frequency (MAF) among the 20 sequenced animals, whether the SNP had previously been validated (or, if not validated, SNPs predicted to be segregating in both breeds were preferred), conservation of SNP flanking sequences in the six species listed previously, and the predicted presence of transcription or other regulatory motifs using the Transfac public database for gene regulation (http://gene-regulation.com/pub/databases.html) and with Illumina design scores >70 (28).

DNA was extracted from tissue from each animal using the Puregene DNA extraction kit following the manufacturer's instructions (Gentra, Minneapolis, MN). DNA was quantified using a NanoDrop1000 spectrophotometer (ThermoScientific, Wilmington, DE) and then genotyped at Igenix (Seattle, WA) using the custom array. Genotypes were called using Illumina's BeadStudio (v3.2.23) software, and samples were removed from the analysis when more than 10% of genotypes failed. After quality control, 210 Holstein cattle (90 cases and 120 controls) and 56 Jersey cattle (15 cases and 41 controls) remained for analysis. SNPs were removed if the MAF was <1% (*n* = 39 in Holstein; *n* = 9 in Jersey), if SNP call rates were <90% (*n* = 7 in Holstein; *n* = 30 in Jersey), or if the SNPs failed the Hardy–Weinberg equilibrium test (*P* < 0.001; *n* = 6 in Holstein; *n* = 4 in Jersey). After filtering, 44 SNPs remained for analysis in Holsteins and 53 in Jerseys.

To test for population stratification between the cases and controls prior to the association analysis, multi-dimensional scaling (MDS) plots were constructed using PLINK (version 1.07) in the R statistical environment for the Holstein and Jersey cattle populations (29). No population stratification was detected among the Holstein or Jersey cattle populations.

### Association Analysis

An allelic chi-square test was performed using PLINK v1.07 between SNPs to identify loci associated with *MAP* infection (29). Two separate analyses were conducted; the first compared cases and controls within the Holstein populations and the second compared cases and controls within the Jersey population. A significance threshold for the association analysis of *P* < 0.05 was used after 10^6^ permutations comparing each observed test statistic against the maximum of all permuted statistics over all SNPs for each single replicate. The linkage disequilibrium levels between SNPs were computed using the D' function in Haploview 4.2 (30).

### Functional Analyses

#### Cell Culture, Transfection, and Dual Luciferase Reporter Assay

Human embryonic kidney (HEK293) cells were cultured in Dulbecco's modified eagle medium (DMEM) supplemented with 10% fetal bovine serum (FBS), 100 units/ml penicillin, and 100 μg/ml streptomycin. Cells were maintained at 37°C in a humidified atmosphere of 95% air and 5% CO_2_. HEK293 cells were seeded at 10^5^ cells per well in a 24-well plate the day before transfection and co-transfected with 0.8 μg of the luciferase reporter construct and 0.1 μg of pRL-TK (*Renilla* luciferase) plasmid. Thirty hours after transfection, firefly and *Renilla* luciferase activities were measured consecutively using a dual Luciferase assay kit (Promega, Madison, WI). *Renilla* luciferase values were normalized to firefly and the ratio of *Renilla/*firefly values was reported. Each experiment was carried out more than three times in triplicate (repeatability = 0.93).

#### Electrophoretic Mobility Shift Assays

Eighteen significant SNPs from the association analysis were further investigated using EMSAs. Nuclear extracts were prepared from cattle ileo-cecal lymph nodes using NE-PER Nuclear and Cytoplasmic Extraction Reagents (Pierce, Rockford, IL) according to the manufacturer's instructions. The EMSAs were performed using the LightShift Chemiluminescent EMSA kit (Pierce, Rockford, IL) according to the manufacturer's instructions. Double-stranded oligonucleotide probes consisting of 31 bp of sequence complementary to each allele were synthesized (Supplementary Table 1). Probes were pre-incubated with poly (dI-dC), a competitor for nonspecific DNA binding proteins, for 2 h at room temperature and then incubated with 20 μg of nuclear extract for 20 min at room temperature. The products were then separated by electrophoresis on a 6% non-denaturing polyacrylamide gel with 0.5× tris-borate-EDTA buffer (pH 8.3). The protein–oligonucleotide complexes were visualized by auto-radiography. For competition studies, unlabeled oligonucleotide probes of 5- to 125-fold excess concentration were pre-incubated with the nuclear extract before the biotin-labeled probes were added. All EMSAs were repeated at least three times to assess the reproducibility of the observed band shifts (*r* = 1.0).

#### 3′ MicroRNA Analysis of EDN2 mRNA Stability

The *MAP* infection-associated SNP272 *(rs109490418*) is located within the 3′ UTR of *EDN2*, 189 bp from the transcription stop site. Putative target sites for microRNA in the 3-UTR around SNP272 were screened using Targetscan (www.targetscan.org) to investigate their potential involvement in mRNA stability. microRNAs were investigated for their role in mRNA stability as SNPs within microRNAs or their binding sites in the 3′ UTR have been demonstrated to influence regulation of gene expression (31). This occurs through the binding of microRNA to the 3′ UTR of mRNA in conjunction with other RNA binding proteins that are responsible for the localization, translation, or degradation of mRNA with the cell (32, 33). Two microRNAs (bta-miR-2339 and bta-miR-1197) were predicted within the 600-bp 3′ UTR of *EDN2* and were investigated using EMSA.

Within the 3′ UTR, bta-miR-2339 was located between 409 and 415 bp while bta-miR-1197 was located 413 to 419 bp from the *EDN2* transcription start site. To determine if the alleles at this site influenced mRNA stability, genomic DNA from two cattle that were homozygous for SNP272 for either the A or G allele were used as the PCR template for the analysis. Sequences for the amplified fragments of 31 bp are in Supplementary Table 2. The 3′ PCR amplified DNA was cloned into the XhoI and NotI multiple cloning sites distal to the *Renilla* luciferase coding region of the psiCHECK-2 vector (Promega, Madison, WI). microRNA precursors (bta-miR-1197 and bta-miR-2339) and a negative control were purchased from Ambion (Austin, TX). HEK293 cells were co-transfected with 50 nmol/L microRNA and 200 ng of psiCHECK-2 constructs, using Lipofectamine 2000 (Invitrogen, Carlsbad, CA). The assay for firefly and *Renilla* luciferase activities were as described above in the “*Cell culture, transfection, and dual luciferase reporter assay*” subsection. The two alternative allele SNP272 sequences were confirmed by sequencing both cloned strands. Results were assessed using a two-tailed *t* test with a significance threshold of *P* < 0.05.

#### 5′ Region Analysis of EDN2

Since SNPs located in the 5′ region of a gene or in a promoter region could alter gene expression through the modification of transcription binding factors, enhancers, or suppressors, any SNP that was confirmed to exhibit differential allelic binding through EMSA was further investigated to determine the alleles' impact on *EDN2* expression (*n* = 2). As SNP208 (*rs110287192*) and SNP105 (*rs109651404*) were confirmed to have different allele binding affinities by EMSAs, both were further examined to determine if there were allele-specific *EDN2* expression differences. SNP208 is found 671 bases 5′ of the transcription start site for *EDN2* located at 104,700,352 (ARS-UCD 1.2). This SNP was part of a 718-bp construct of the *EDN2* promoter (104,699,956–104,700,673 bp) flanked by XhoI and HindIII restriction endonuclease sites and inserted into a pGL3-basic vector (Supplementary Figure 1A; Promega, Madison, WI). Constructs were created containing each of the two SNP208 alleles.

SNP105 is located 11 kb 5′ to the transcription start site of *EDN2*. Considering the substantial physical distance between SNP105 (G/A) and *EDN2*, luciferase assays were not performed on this SNP alone. Instead, it was co-transfected into pGL3-basic vectors that contained SNP208 (G/T) to determine if allele-specific interactions between the SNP105 and SNP208 influenced *EDN2* expression (Supplementary Figure 1B). A 31-bp (104,689,846–104,689,876 bp) PCR fragment containing either of the SNP105 alleles was amplified from individuals with DNA that were homozygous for the allele. The amplified products for each allele were separately cloned into the plasmids that contained SNP208 and a portion of the *EDN2* promoter. The sequences of the cloned PCR fragments were verified by sequencing both the sense and antisense strands of the cloned fragments. Relative luciferase activities were compared between the four plasmid constructs containing the four different allelic combinations: (1) SNP105-G and SNP208-G, (2) SNP105-G and SNP208-T, (3) SNP105-A and SNP208-G, and (4) SNP105-A and SNP208-T. Luciferase results were analyzed using a two-tailed *t* test, and interactions with *P* < 0.05 were considered significant.

## Results

### Association Analyses

**Figure 1:**
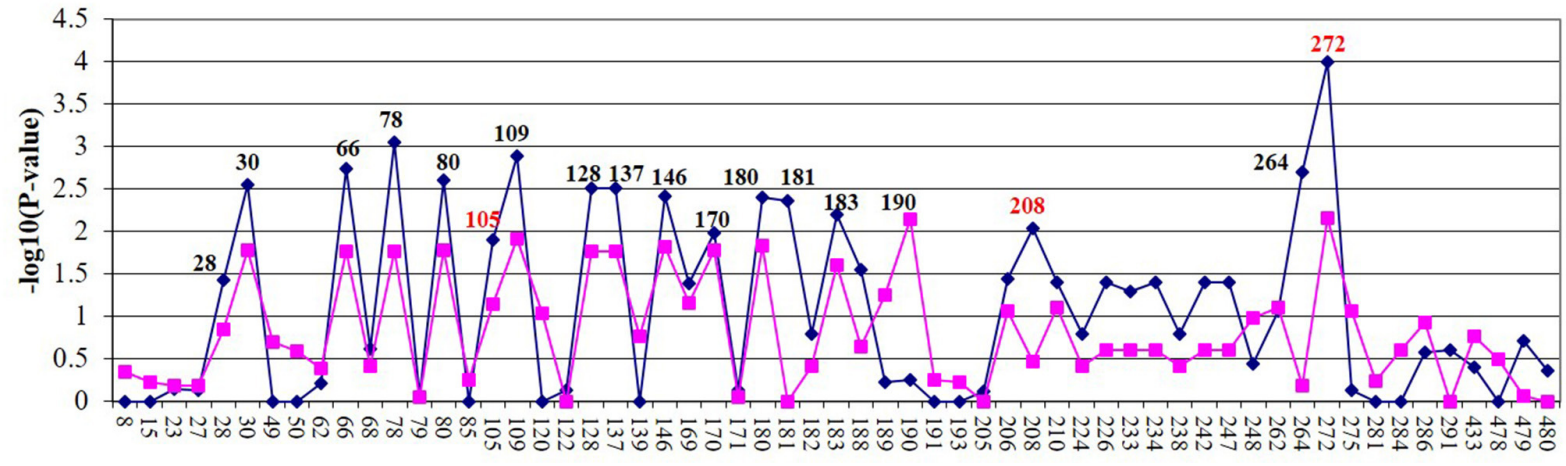
Fine mapping of Holstein (blue) and Jersey (lavender) cows of 70-kb region on BTA3. SNP name/number are listed on the *X* axis. Significant SNPs (*P* < 0.05) were examined by electrophoretic mobility shift assay (EMSA). SNPs in red were further evaluated using luciferase assays.

Allelic association tests were conducted to determine if there was an association between *MAP* tissue infection and the selected SNPs. The allelic chi-square test identified 24 SNPs (*P* < 0.05) associated in the Holstein population and 13 SNPs in the Jersey population (Figure 1). The most significant SNP in both populations was SNP272 (Holstein: *P* < 9.9 × 10^−5^; Jersey: *P* < 6.8 × 10^−3^). The 18 SNPs most strongly associated with *MAP* tissue infection across both breeds were selected for further functional analyses. Apart from a single SNP (SNP190, no rs#), all significant SNPs were in strong linkage disequilibrium with each other (*r*^2^ > 0.9). Additional investigation determined that SNP272 was located within the 3′ UTR of *EDN2* while the remaining SNPs were located 5′ of *EDN2*.

### Electrophoretic Mobility Shift Assays

**Figure 2:**
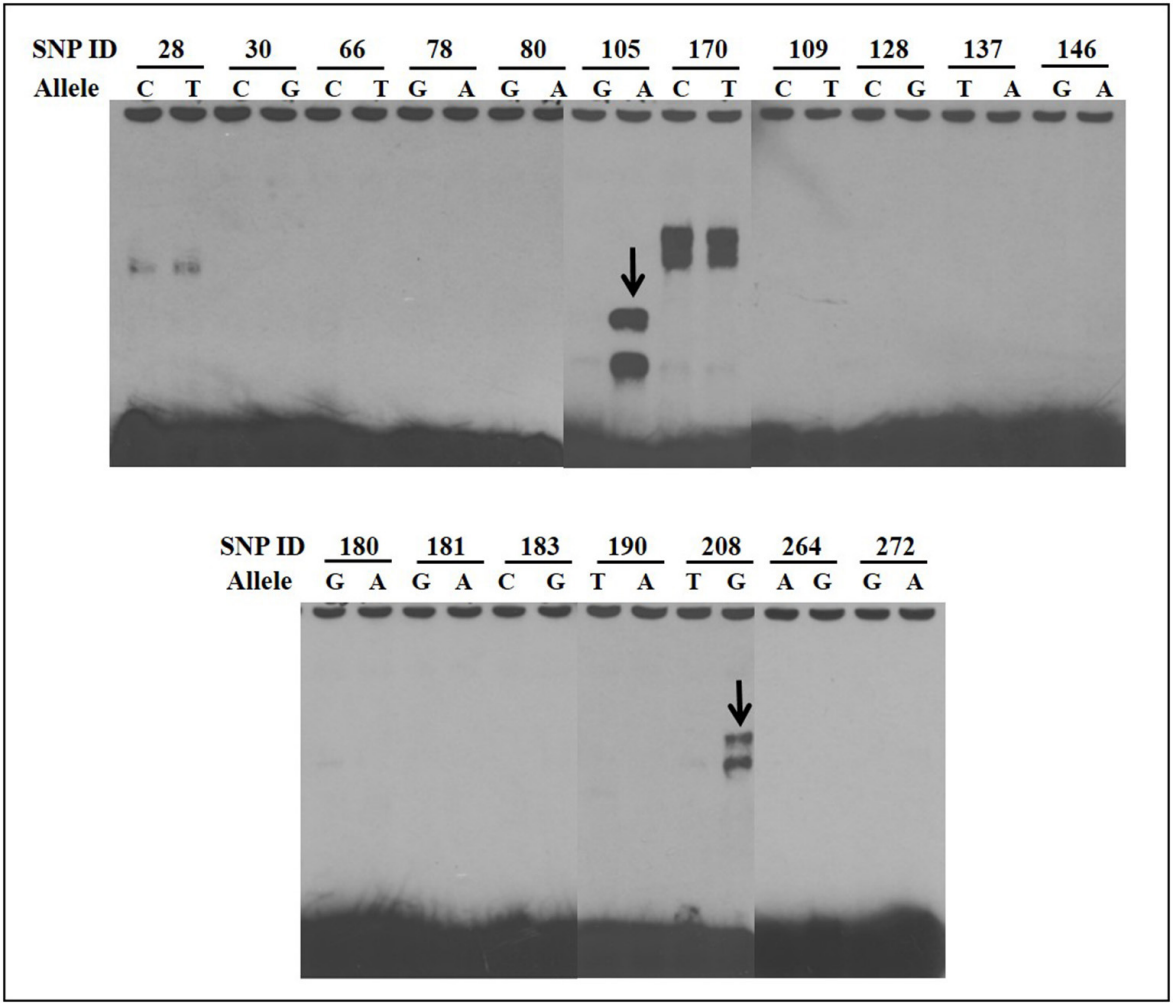
Electrophoretic mobility shift assays (EMSAs) to screen for functional SNPs showing differential affinities to nuclear proteins between susceptible and non-susceptible alleles. Differential bands for SNP105 and SNP208 are indicated with arrows. All EMSAs were performed in triplicate with the results from a single replicate shown in this figure.

**Figure 3:**
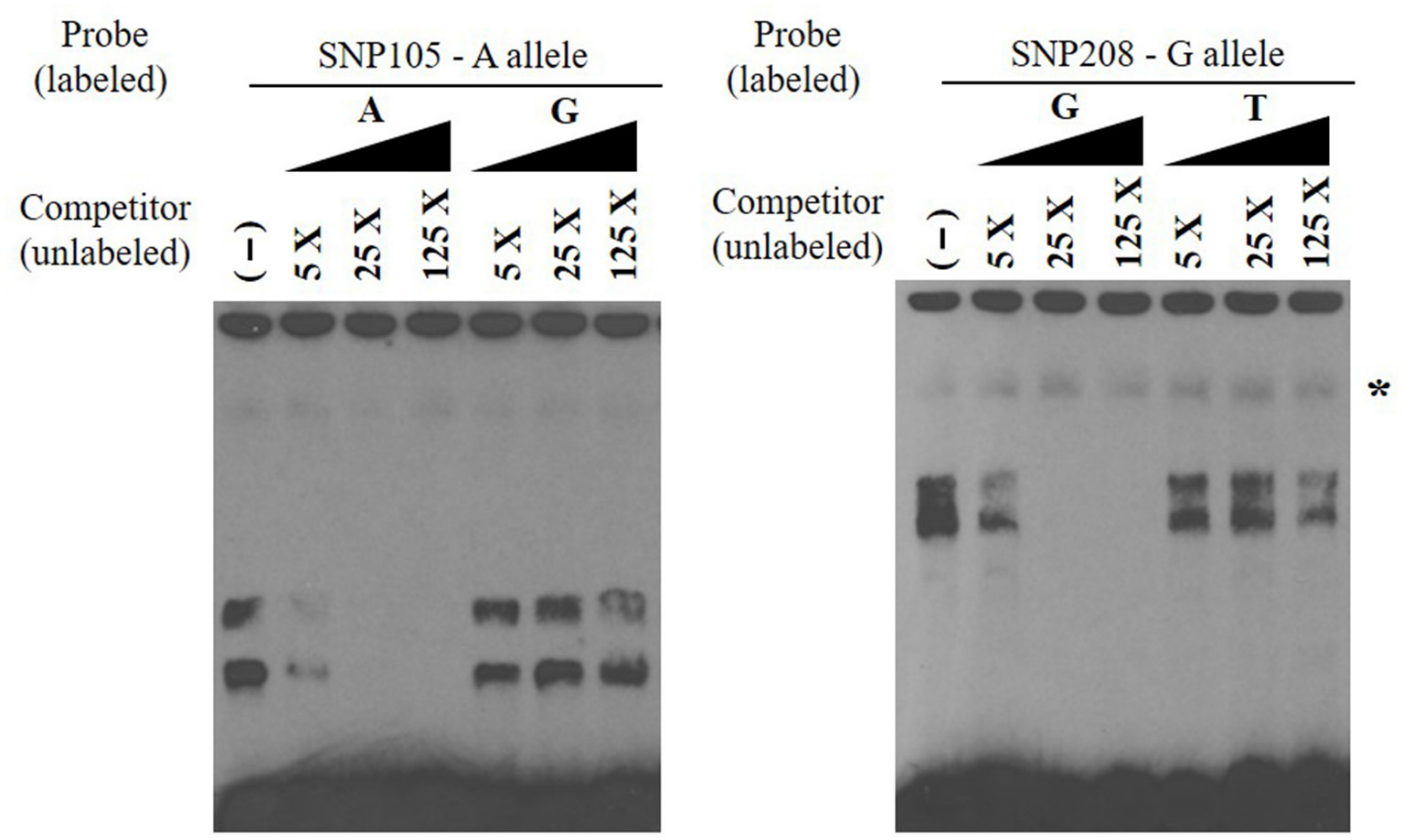
Electrophoretic mobility shift competition assay for SNP105 (**left**) and SNP208 (**right**). DNA–protein complexes were competed away in a concentration-dependent manner by unlabeled oligonucleotide with A allele for SNP105 and with allele G for SNP208 (non-specific binding was indicated with star). All EMSAs were performed in triplicate with the results from a single replicate shown in this figure.

EMSAs were used to determine if allelic variants at the 18 most significant SNPs from the association analysis had a functional role in binding ileo-cecal lymph node nuclear proteins, since binding proteins have a known role in controlling the production of mRNA within a cell (34). Only 2 of the 18 SNPs (SNP105 or *rs109651404* and SNP208 or *rs110287192*) exhibited differential nuclear protein binding affinities between alleles (Figure 2). Additional competition EMSAs were conducted for these two SNPs (Figure 3), confirming the differential binding affinity of alleles.

### 3′ MicroRNA Analysis of mRNA Stability

**Figure 4:**
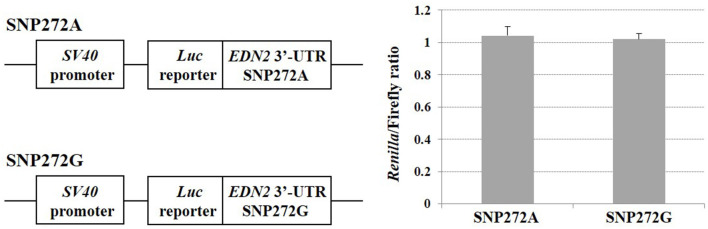
SNP272 effect on mRNA stability. HEK293 cells were transfected with *EDN2*-3′-UTR luciferase constructs (SNP272 A/G). Luciferase activities (in triplicates) were measured 24 h post-transfection. *Renilla* luciferase activities were normalized against firefly luciferase activities, and mean normalized *Renilla* luciferase activities (± SD) from three independent experiments were determined. There was no difference (*P* > 0.05) in luciferase activities between the A and G alleles at SNP272.

**Figure 5:**
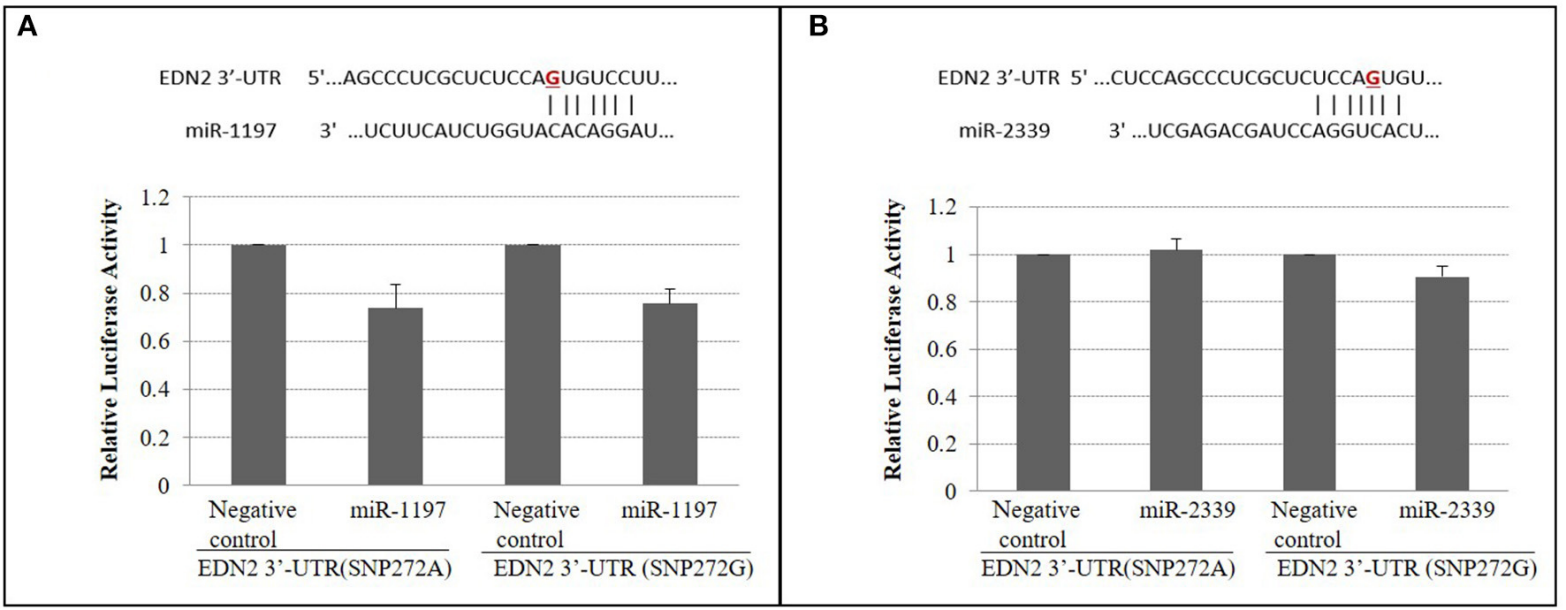
**(A)** SNP272 is not responsible for bta-miR-1197's targeting of *EDN2* 3-UTR (*P* > 0.05). miR-1197 and its predicted seed binding site in the 3′-UTR of *EDN2* (**top**). SNP272 is highlighted in red. **(B)** bta-miR-2339 does not target 3′-UTR of *EDN2* (*P* > 0.05). miR-2339 and its predicted seed binding site in the 3′-UTR of *EDN2* (**top**). HEK293 cells were co-transfected with *EDN2*-3′-UTR luciferase constructs (SNP272 A/G) and microRNA precursors (miR-1197 or miR-2339). microRNA negative control oligonucleotides were used as negative controls. Luciferase activities (in triplicates) were measured 24 h after transfection. *Renilla* luciferase activities were normalized against firefly luciferase activities, and mean normalized *Renilla* luciferase activities (±SD) from three independent experiments were determined and expressed relative to control values.

SNPs within microRNAs or their binding sites in the 3′ UTR have been demonstrated to influence regulation of gene expression (31). No significant change of luciferase activities was observed (*P* > 0.05) (Figure 4) when A vs. G alleles were compared for SNP272 luciferase constructs. Similarly, no significant changes in reporter activities were identified (*P* > 0.05; Figure 5) for the allele-specific effects of SNP272 when the binding of microRNA (bta-miR-2339 or bta-miR-1197) to the 3′ UTR was compared. These results indicate that the microRNAs are not influenced by SNP272 variation and that this variant is not influencing mRNA stability through alterations of the microRNA binding sites.

### 5′ Region Analysis

**Figure 6:**
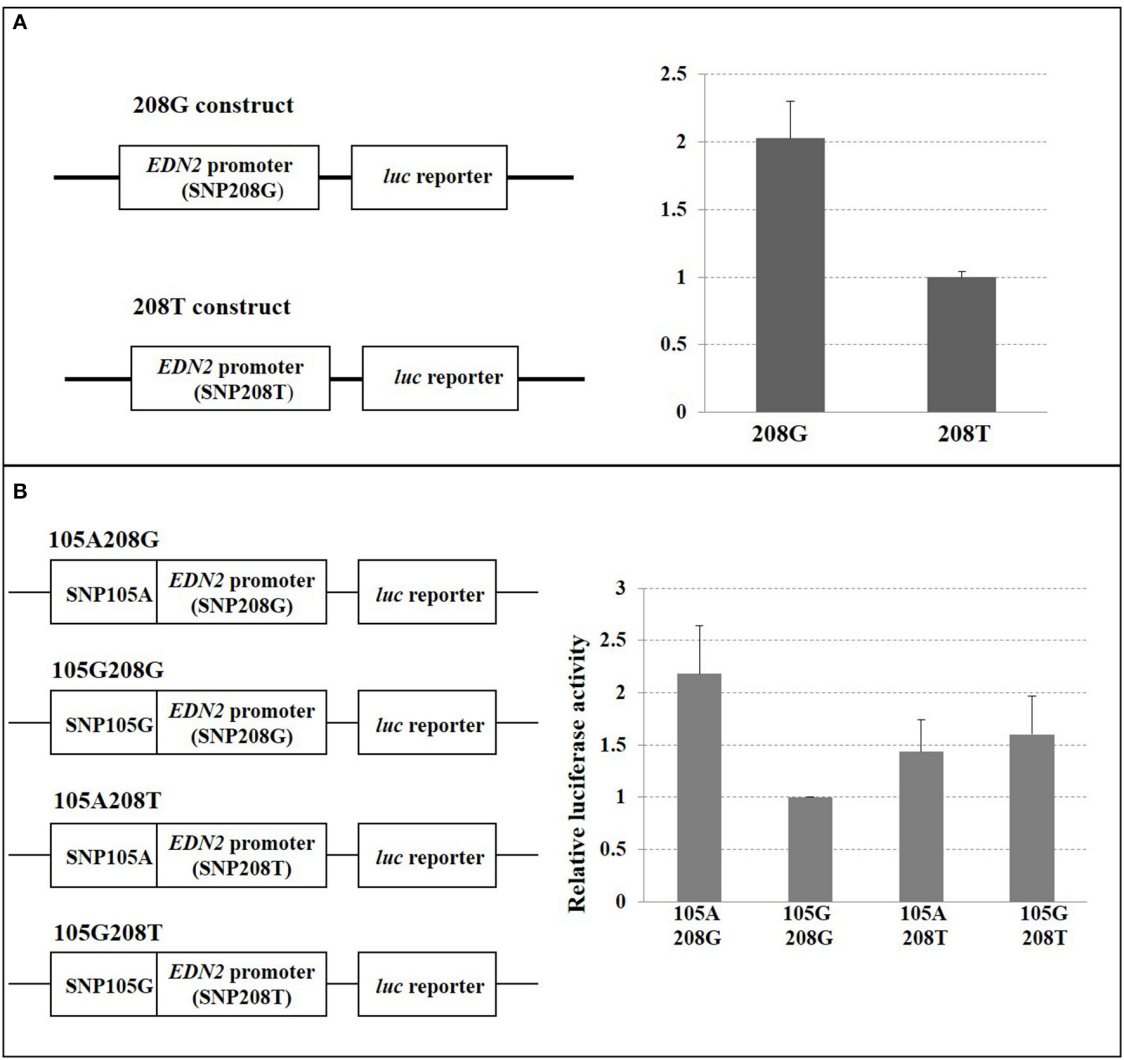
**(A)** SNP208 Luciferase reporter assay. HEK293 cells were transfected with *EDN2* promoter-pGL3 reporter constructs containing either G or T at the location of SNP208. The relative luciferase activities were calculated and expressed as mean ± SD. The relative luciferase activity of the genotype G at SNP208 was significantly higher (*P* = 0.01) compared with that of the genotype T. **(B)** SNP105 and SNP208 interaction. HEK293 cells were transfected with *EDN2* promoter-pGL3 reporter constructs containing different combination of SNP105(A/G) and SNP208(G/T). The relative luciferase activities were calculated and normalized to construct 105G/208G. The mean of relative luciferase activity (±SD) from three independent experiments was shown. The relative luciferase activity of the G genotype at SNP208 and the A genotype at SNP105 was significantly higher (*P* = 0.02) than genotype combinations.

When SNP208 and SNP105 were investigated, the G allele of SNP208 had twice the luciferase activity as the T allele of SNP208 when paired with the *EDN2* promoter (Figure 6A; *P* = 0.01). The increased luciferase activity of the G allele at SNP208 when paired with the *EDN2* promoter was further supported by the results from the luciferase assay on the co-transfected plasmids containing alleles for SNP208 and SNP105. When the G allele for SNP208 and the A allele for SNP105 were paired with the *EDN2* promoter, there was a significant increase in luciferase activity (*P* = 0.02) compared to all other allelic combinations with the *EDN2* promoter (Figure 6B).

## Discussion

Numerous studies have attempted to identify the genetic variation involved in susceptibility to *MAP* infection. Various approaches have been applied to identify genes and chromosomal regions associated with *MAP* infection, including genome-wide association, candidate gene, and linkage analyses. An initial genome-wide association study (15) and subsequent fine-mapping study (18) revealed a region of 10.6 kb on BTA3 associated with *MAP* infection. The current study expanded on these two studies by identifying 18 SNPs associated with *MAP* tissue infection through re-sequencing and fine-mapping in both Holstein and Jersey breeds when the region between *EDN2* and *HIVEP3* was evaluated. Of the 18 SNPs tested in the EMSA analyses, three (SNP272, SNP208, and SNP105) showed evidence of binding nuclear proteins. This study provides the basis for further investigations to identify the proteins that bind these regions and the mechanism used to regulate *EDN2*. This investigation is needed for a full understating of the role of these SNPs in regulating *EDN2* and *Map* infection.

An *in silico* analysis of the 18 SNPs associated with *MAP* infection did not provide evidence that the SNPs were within transcription factor binding sites using the PROMO transcription regulatory element search tool (35, 36). However, given the level of sequence conservation across species in this region and the limited availability of annotated regulatory regions in cattle, further investigation was warranted. The regulatory function of SNP208 and SNP105 was confirmed through the use of luciferase assays, while the variant alleles at SNP272 did not regulate *EDN2*. The favorable allele of SNP208 and SNP105 increased luciferase activity alone and when coupled together, indicating that these two SNPs may synergistically regulate *EDN2* expression. Previous studies have also used luciferase assays to determine if SNPs located within microRNA binding sites were associated with certain disease traits in the 3′ UTR of genes. For example, Zou et al. (33) performed luciferase assays on 10 SNP located within microRNA binding sites in the 3′ UTR of genes associated with oxidative damage and age-related cataracts. They found that an allele in the 3′ UTR of the XPC gene differed in luciferase reporter gene expression and was linked to an increased risk of nuclear type of age-related cataracts. Similar to the results of Zou et al. (33), this study also identified that SNPs within microRNA binding sites in the 3′ UTR may be associated with disease traits.

The role of *EDN2* in *MAP* infection may be through its interaction with macrophages in providing intestinal immunity or in its role in the contraction and permeability of the intestine. During initial infection, *MAP* enters the host most commonly through the fecal–oral route where it is then transported across the intestinal epithelial barrier and into the host macrophages. Once *MAP* infects the host macrophages, it is able to avoid the host's phagosome–lysosome response (37). While the mechanism that *MAP* employs to avoid the phagosome–lysosome response is unknown, other mycobacteria are capable of avoiding the phagosome–lysosome interaction within macrophages through the secretion of phosphatase, an acid that can arrest phagosomal maturation (38). Several studies have indicated that *EDN2* produces a chemoattractant for macrophages, such that an increased expression of *EDN2* attracts macrophages to a given area (39). While *EDN2* is commonly associated with female reproduction and ovulation (40–42), it is also present in the gastrointestinal tract of multiple species where it is highly expressed (Human Protein Atlas available from https://www.proteinatlas.org/ENSG00000127129-EDN2/tissue/small+intestine) (43, 44). A study in mice by Takizawa et al. (43) found that localization of EDN2 was predominantly observed in the epithelial cells of the basal membrane and that EDN2 could be secreted into the lamina propria and the dome region of Peyer's patch, suggesting a role in modulating mucosal defense. Previous work has indicated that Peyer's patches in the small intestines of calves and kid goat play a key role in mediating *MAP* uptake across the intestinal barrier (45, 46).

In addition to its potential role in intestinal mucosal defense in mice, *Edn2* also has a function in intestinal architecture. Bianchi et al. (47) found that in mouse intestinal villi, EDN2 exhibited a gradient localization, with increased presence near the base or crypt of the villi. The researchers postulated that the increased localization of EDN2 near the crypts of intestinal villi was correlated with smooth muscle contraction (mobility) of the villi, contributing to the overall structure of the intestinal villi. It was also suggested that the gradient expression of EDN2 might be associated with villi permeability through changes in the fibroblast network (47).

Given the roles that *EDN2* has in macrophage signaling, intestinal mucosa, intestinal structure, and how important these functions are during *MAP* infection, the hypothesis that potential causal mutations associated with *MAP* tissue infection lie near or within the gene is supported. The identification of SNP208 to be associated with *MAP* infection across breeds in different parts of the world provides further evidence that this region surrounding *EDN2* is associated with the disease. Çinar et al. (48) also investigated SNP272, SNP208, and SNP105 for their roles in *MAP* infection and identified SNP208 to be associated with *MAP* infection in East Anatolian Red crossbred cattle, Anatolian Black crossbred cattle, and Holstein cattle. Çinar et al. (48) found that *MAP* infection differed between animals with different genotypes at SNP208 (*P* < 0.05) where cattle with the TG or GG genotypes were more often controls and cattle with TT genotypes were cases, suggesting that animals with the G may be less susceptible to *MAP* infection. Çinar et al. (48) did not identify any differences in *MAP* infection for SNP105 or SNP272.

The use of a SNP identified across breeds to be associated with *MAP* infection for genomic selection would be advantageous as it would be predictive for disease susceptibility in multiple breeds. The use of a causal variant for disease is even more desirable as the accuracy of prediction would not decay over time. Although SNP208 has not yet been proven to be a causal variant for *MAP* infection, these results are supportive of its role in *MAP* infection and suggest that it may be of value for genomic selection to reduce disease. The selection of cattle less susceptible to *MAP* infection could reduce the prevalence of bovine paratuberculosis and lower the cattle suffering and the economic costs associated with it.

## Data Availability Statement

The data presented in the study are deposited in the CattleQTLdb repository, and can be found at: https://www.animalgenome.org/QTLdb/supp/?t=TaEg1A5OgI.

## Ethics Statement

The animal study was reviewed and approved by Washington State University Institutional Animal Care and Use Committee (Study# 4073). Written informed consent for participation was not obtained from the owners because for the Jersey population, verbal consent was obtained from dairy owners prior to sample collection which occurred post mortem. For the Holstein population, these samples were collected from the Regional Dairy Quality Management Alliance (RDQMA) study herds for other analyses.

## Author Contributions

HN, JT, and SW designed the study. HN, RZ, and ES collected the samples. SW and CT provided sequencing and transcription factor data. ZW, RZ, ES, MN, JK, and BC performed the experiments. JK wrote and edited the manuscript. HN reviewed and edited the manuscript. All authors read and approved the final manuscript.

## Conflict of Interest

The authors declare that the research was conducted in the absence of any commercial or financial relationships that could be construed as a potential conflict of interest.
