# Supplementary material for: Functional Variants Surrounding Endothelin 2 Are Associated With Mycobacterium avium Subspecies paratuberculosis Infection
Source: Front Vet Sci. 2021 May 5;8:625323. doi: 10.3389/fvets.2021.625323 (PMC8131860; doi:10.3389/fvets.2021.625323)
Supplement: Supplementary file 1 [file Table_1.DOCX]

**Supplementary Table 1.** Sense and antisense oligonucleotides for 18 most significant SNP associated with *Mycobacterium* *avium* ssp. *paratuberculosis* (*MAP*) infection.

| SNP Name^1^ (*rs#*) | Location^2^ | Oligonucleotides^3^ |
| --- | --- | --- |
| SNP28**^*^**  (*rs211116632*) | 105,279,109  (104,680,796) | AGGATTACTCAGACA**C/T**AGCCTGGGAAAACCG  (CGGTTTTCCCAGGCT**G/A**TGTCTGAGTAATCCT) |
| SNP30**^*^**  (*rs109206971*) | 105,279,358  (104,681,045) | GCGAGGGCTGCACTG**C/G**GGGAGTCAGTTTATC  (GATAAACTGACTCCC**G/C**CAGTGCAGCCCTCGC) |
| SNP66**^*^**  (*rs208992208*) | 105,284,915  (104,686,602) | GACCTCGTTGTCCAG**C/T**ACGAGACTCAGGGGA  (TCCCCTGAGTCTCGT**G/A**CTGGACAACGAGGTC) |
| SNP78**^*^**  (*rs210788783*) | 105,286,393  (104,688,080) | GATTTGGTTCAACAA**G/A**CCTTCAAACCACCTT  (AAGGTGGTTTGAAGG**C/T**TTGTTGAACCAAATC) |
| SNP80**^*^**  (*rs208212914*) | 105,286,650  (104,688,337) | GATCTGTCCTGTGTC**G/A**GGGTCAGAGGCTTGC  (GCAAGCCTCTGACCC**C/T**GACACAGGACAGATC) |
| SNP105**^*^**  (*rs109651404*) | 105,288,174  (104,689,861) | CAAAAGGCTCCCAGT**G/A**TGTCTTCCAGGTGCT  (AGCACCTGGAAGACA**C/T**ACTGGGAGCCTTTTG) |
| SNP109**^*^**  (*rs210275942*) | 105,288,737  (104,690,424) | TGGCCTCCTGGCTGG**C/T**GACGGCTGCCTCTCT  (AGAGAGGCAGCCGTC**G/A**CCAGCCAGGAGGCA) |
| SNP128**^*^**  (*rs208027625*) | 105,291,541  (104,693,228) | GGTCTGACACCAGAA**C/G**CAGAGACCTTAGCTA  (TAGCTAAGGTCTCTG**G/C**TTCTGGTGTCAGACC) |
| SNP137**^*^**  (*rs210213005*) | 105,291,983  (104,693,670) | CCTTTTTGATGGGCC**T/A**TGAGTTTGGGCAAAA  (TTTTGCCCAAACTCA**A/T**GGCCCATCAAAAAGG) |
| SNP146**^*^**  (*rs208665231*) | 105,292,844  (104,694,531) | GGCAGCCAGAGCAGC**G/A**GAGCCTCAGAGATTC  (GAATCTCTGAGGCTC**C/T**GCTGCTCTGGCTGCC) |
| SNP170**^*^**  (*rs209417404*) | 105,295,131  (104,696,819) | AAGGGGGTGTCCTCC**C/T**GGGGAAGCCGCAAAG  (CTTTGCGGCTTCCCC**G/A**GGAGGACACCCCCTT) |
| SNP180**^*^**  (*rs207735541*) | 105,296,189  (104,697,877) | TACACTGGTGTGCAG**G/A**AAAGGGTGAGTCTGG  (CCAGACTCACCCTTT**C/T**CTGCACACCAGTGTA) |
| SNP181**^*^**  (*rs381771846*) | 105,296,223  (104,697,911) | AGTAGCCCGGAGAGT**G/A**TGTCCAGCCCATCAC  (GTGATGGGCTGGACA**C/T**ACTCTCCGGGCTACT) |
| SNP183**^*^**  (*rs210989163*) | 105,296,667  (104,698,355) | GAGGCATAGAGGTGA**C/G**TGTCGGGGCCCAGGC  (GCCTGGGCCCCGACA**G/C**TCACCTCTATGCCTC) |
| SNP190**^*^**  (n/a) | 105,297,461  () | TTTTACTTTCTCAAA**T/A**CCAAGTTCTTTCCTT  (AAGGAAAGAACTTGG**A/T**TTTGAGAAAGTAAAA) |
| SNP208**^*^**  (*rs110287192*) | 105,298,664  (104,700,352) | CTTTTGGTGTACATA**T/G**CCACAAGCCACCTTT  (AAAGGTGGCTTGTGG**A/C**TATGTACACCAAAAG) |
| SNP264**^+^**  (*rs132750460*) | 105,304,227  (104,705,915) | AGGTCAACAGCCTGA**A/G**GAGCCACAGGGATAG  (CTATCCCTGTGGCTC**T/C**TCAGGCTGTTGACCT) |
| SNP272**^^^**  (*rs109490418*) | 105,305,070  (104,706,758) | AGCCCTCGCTCTCCA**G/A**TGTCCTTATTCTCCC  (GGGAGAATAAGGACA**C/T**TGGAGAGCGAGGGCT) |

^1^Symbol next to SNP name indicates where the SNP is located in relation to *EDN2*: 5’ flanking region = **^*^**, intron = **^+^**, and 3’ untranslated region = ^^^.

^2^Base pair locations of SNP are listed in based on the UMB 3.1 Assembly with the new ARS Assembly coordinates in parentheses.

^3^Thirty one bp oligonucleotide are listed for sense and anti-sense (in parentheses) sequences. The reference and alternative alleles for each SNP is listed in bolded red letters.
