# Supplementary material for: Functional Variants Surrounding Endothelin 2 Are Associated With Mycobacterium avium Subspecies paratuberculosis Infection
Source: Front Vet Sci. 2021 May 5;8:625323. doi: 10.3389/fvets.2021.625323 (PMC8131860; doi:10.3389/fvets.2021.625323)
Supplement: Supplementary file 2 [file Table_2.DOCX]

**Supplementary Table 2.** Sense and antisense oligonucleotides for SNP associated within 3’-UTR of endothelin 2 associated with *Mycobacterium* *avium* ssp. *paratuberculosis* (*MAP*) infection.

| SNP Name (*rs#*) | Location^1^ | Oligonucleotides |
| --- | --- | --- |
| SNP272  (*rs109490418*) | 105,305,070  (104,706,758) | ATCTCGAGCTCTCGACTCTGGGAGAACTTTGGGAAG (ATGCGGCCGCATTATTTTGTTGTTTATTACAAACACAAGTTCGCA) |

^1^Base pair locations of SNP are listed in based on the UMB 3.1 Assembly with the new ARS Assembly coordinates in parentheses.
