# Supplementary figures and images for: Functional Variants Surrounding Endothelin 2 Are Associated With Mycobacterium avium Subspecies paratuberculosis Infection
Source: Front Vet Sci. 2021 May 5;8:625323. doi: 10.3389/fvets.2021.625323 (PMC8131860; doi:10.3389/fvets.2021.625323)

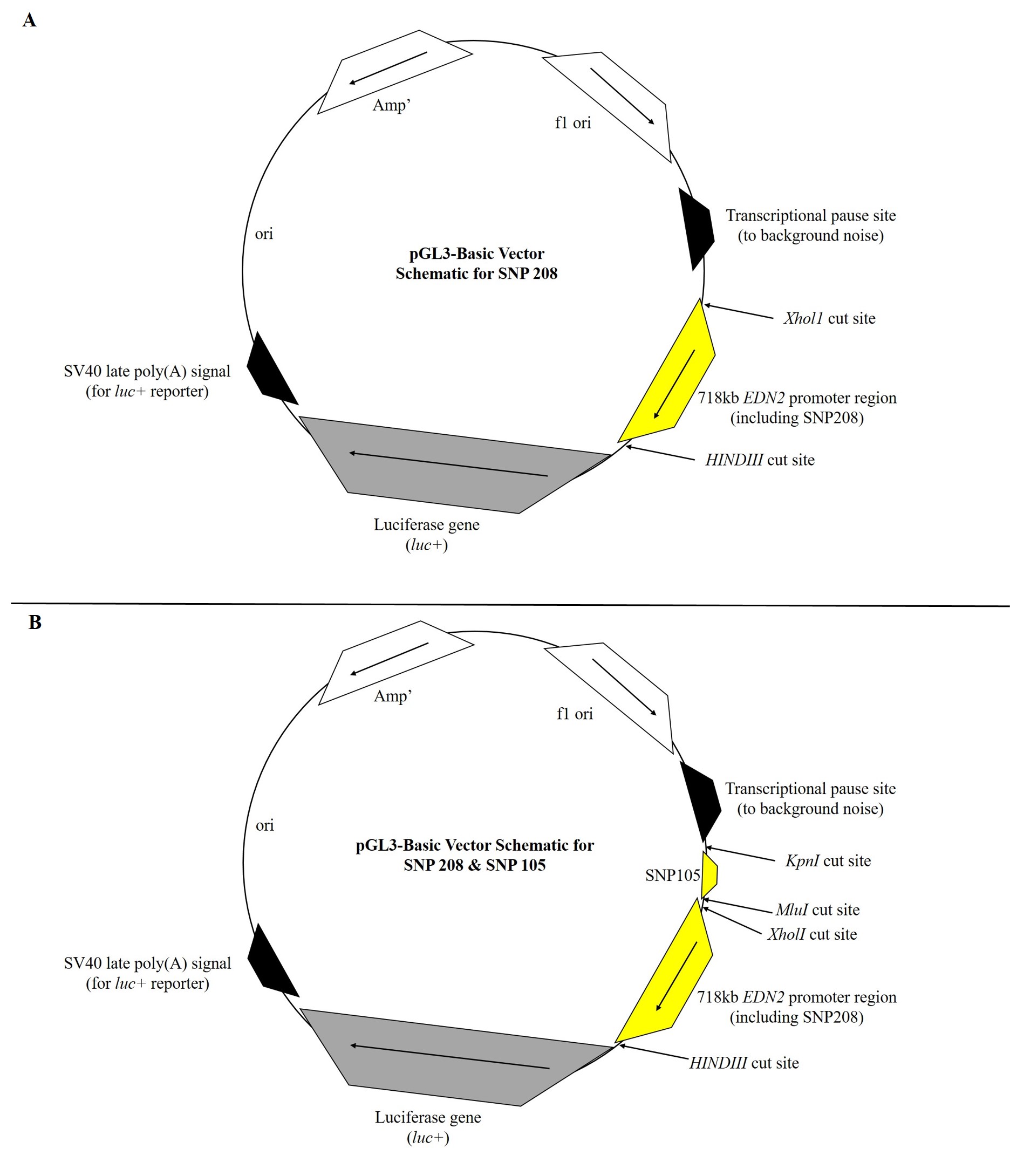

Supplement: Supplementary file 3 [file Image_1.JPEG]
